# Late intervention for type II endoleak is not determined by early sac diameter or volume changes after EVAR

**DOI:** 10.2478/raon-2024-0056

**Published:** 2024-11-28

**Authors:** Bernard Sneyers, Viktor Verbraeken, Annouschka Laenen, Walter Coudyzer, Hozan Mufty, Sabrina Houthoofd, Inge Fourneau, Geert Maleux

**Affiliations:** Department of Radiology, University Hospitals KU Leuven, Leuven, Belgium and Department of Imaging & Pathology, KU Leuven, Leuven, Belgium; Department of Public Health and Primary Care, Leuven Biostatistics and Statistical Bioinformatics Centre, Leuven, Belgium; Department of Vascular Surgery, University Hospitals KU Leuven, Leuven, Belgium

**Keywords:** endovascular aneurysm repair, type II endoleak, diameter, volume, changes in measurement

## Abstract

**Background:**

To compare the diagnostic accuracy and predictive value of aneurysm sac volume measurement versus maximum diameter measurement of abdominal aortic aneurysm sac after endovascular aneurysm repair (EVAR) in patients with type II endoleak.

**Patients and methods:**

Retrospective study on a cohort of 103 patients who presented with a type II endoleak after EVAR for infrarenal abdominal aortic aneurysm. Maximum diameter and volumetric measurements were calculated on computed tomography follow-up scans at 3 months and 1 year after index surgery. Pearson correlation coefficient was used to determine linear association between diameter and volume; Mann-Whitney U test was used to compare patients with and without later intervention for type II endoleak with regard to diameter and volume change.

**Results:**

The correlation between diameter and volume measurement was high (Rho: 0.890–0.980 with P < 0.0001). In 38 out of 103 patients (37%) with type II endoleak, a later intervention for endoleak management was performed; early diameter (P = 0.097), or volume (P = 0.387) change could not predict risk for later intervention.

**Conclusions:**

Both diameter and volume measurements can be used in the imaging follow-up of patients with endoleak type II after EVAR; however early changes in diameter or volume of the aneurysm sac cannot predict late intervention for type II endoleak.

## Introduction

Endovascular aneurysm repair (EVAR) for infrarenal abdominal aortic aneurysms (AAA’s) has become the preferred treatment option related to the reduced risk of peri- and postoperative morbidity and mortality compared to open surgical repair.^1^ However, persistent growth of the aneurysm sac after EVAR, associated with endoleak, is a risk factor for rupture and further management, including characterisation of the underlying endoleak and subsequent treatment, are mandatory.^2^ Malignant endoleaks, including type I and type III endoleaks, should be promptly treated, once detected; type II endoleaks most probably need additional treatment if associated with persistent sac growth; if not, these endoleaks are considered as benign endoleaks and only need further imaging follow-up.^3^ In case treatment is mandatory, mean time interval between initial EVAR and type II endoleak treatment is > 3 years.^4^ Predictive imaging factors for future need to treat a type II endoleak include endoleak volume, endoleak diameter, number of patent aortic side branch vessels before EVAR and a complex type endoleak pattern.^5,6^ Furthermore, repeated tri-phasic contrast-enhanced computed tomography angiography (CTA) including a high cumulative, radiation dose and intravenous iodized contrast medium administration is needed in case a type II endoleak is detected.

Early detection of volumetric changes in the excluded aneurysm sac in patients with a type II endoleak could be a potential alternative for a better, earlier and more accurate selection of patients with malignant type II endoleak.

However, it is still unclear whether volume measurement or maximum diameter measurement of the excluded aneurysm sac is the most accurate for monitoring sac growth^7,8,9^; in addition, most of imaging studies comparing volume to maximum diameter measurement are dealing with patients with and without endoleaks. In this study, we analyzed the concordance between changes in maximum diameter compared to changes in volume measurement of the excluded aneurysm sac in patients presenting with type II endoleak after EVAR. Finally, we evaluated if early diameter and/or volume changes might be predictive for type II endoleaks associated with later persistent and substantial growth, ultimately requiring treatment.

## Patients and methods

### Study design and inclusion criteria

Patients who underwent an elective EVAR procedure to treat an AAA in the authors’ institution between January 2002 and August 2019 and presenting with a type II endoleak on follow-up CT-imaging at 3 months and 1 year after the index EVAR-procedure, were included in this retrospective study. Patients with concomitant type I and/or type III endoleak were excluded. Patients gave informed consent for the EVAR-procedure and the follow-up CT-imaging and this retrospective study, with number MP11800, was approved by the local Ethics Committee (No. MP11800) from the University Hospitals Leuven, Belgium. Demographics and clinical follow-up data were collected from the patients’ electronic medical records and CT-imaging analysis was performed on a dedicated imaging workstation, connected to the institutional Picture and Archiving Communication System (PACS, Agfa Gevaert, Mortsel, Belgium).

### Computed tomography angiography scan protocol

All CTA-studies were performed on 16-, 64- or 256-row multidetector computed tomography (MDCT) scanners depending on the time period of performing the study. Briefly, patients underwent triphasic MDCT protocol, consisting of unenhanced, arterial and venous phase acquisitions at 3 months and 1 year after the index EVAR-procedure. The contrast-enhanced phases were performed after intravenous injection of a bolus of 100 ml nonionic, iomeprol iodinated contrast medium (Iomeron 350, Bracco, Milano, Italy) at a flow rate of 3 ml/second followed by 25 ml of saline flush at a flow rate of 3 ml/second into an antecubital vein. The start of the arterial phase scan was defined by bolus tracking technique with an attenuation threshold of 130 Hounsfield Units (HU) at the level of the supracoeliac portion of the abdominal aorta. Data acquisition started 6 seconds for the arterial and 80 seconds for the venous phase respectively after reaching the 130 HU threshold. Other scan parameters included: detector collimation of 128 × 0.6 mm, tube kilovoltage of 120 kV, reference mAs of 180 mAs with active CareDose, gantry rotation time of 0.5 seconds and a pitch 0.9, 0.5 for reconstructions 1mm and 3 mm respectively.

Image reconstructions and measurements were performed on a dedicated workstation with postprocessing software (Syngo.via, Siemens Healthcare, Forchheim, Germany). Axial (Ax) diameter is defined as the maximum distance between both outer borders of the aneurysm sac as measured on an axial image; perpendicular (Per) diameter is defined as the maximum distance between the outer border of the aneurysm sac as measured on a reconstructed image, perpendicular on the central lumen line of the abdominal aorta. Aortic sac volume measurements were calculated by semi-automated segmentation from the lowest renal artery to the aortic bifurcation. All measurements were performed in consensus by 2 radiologists with 5 and 25 years of experience in vascular radiology respectively.

### Patients’ follow-up protocol

Patients were followed-up by physical examination and triphasic CTA at 3 months, 1 year after index EVAR and yearly by CTA afterwards, in line with the EUROSTAR follow-up protocol.^10^ Indication for type II endoleak treatment was made in consensus after multidisciplinary discussion, by vascular surgeons and interventional radiologists, involved in the institutional EVAR-program. Treatment was advised if the type II endoleak persist and the maximum diameter of the aneurysm sac increased with > 1 cm compared to the pre-EVAR sac diameter.

### Statistical analysis

Statistical methodology included the Pearson correlation coefficient (ϼ), which was used to determine the strength of the linear association between two continuous variables (Ax / Per diameter and volume); a reliability coefficient less than 0.40 was considered as poor, 0.40–0.59 as fair, 0.60–0.74 as good and 0.75–1.00 as excellent. The Mann-Whitney U test was used to compare patients with and without late intervention for type II endoleak with regards to aneurysm sac diameter and volume change at 3 months and 1 year of follow-up after EVAR. Diameter and volume changes were calculated as both absolute and relative changes. The absolute change was calculated as the second value minus the first value, with a positive number indicating increase and a negative number indicating decrease in sac diameter / volume. The relative change was calculated as the percentage increase or decrease with respect to the first value; e.g. a relative change of 10 indicates a 10% increase. All tests are two-sided and assumed a 5% significance level.

The Kappa coefficient (κ) was calculated as a measure of agreement between two binary variables. Both diameter cut-off values were associated with volume cut-off values. A Kappa-value of 0 indicates no agreement, a value 0.01–0.20 as none to slight, 0.21–0.40 as fair, 0.41–0.60 as moderate, 0.61–0.80 as substantial and 0.81–1.00 as almost excellent agreement.

Analyses have been performed using SAS-software, version 9.4 of the SAS System for Windows (Cary, N-Y, US)

### Data availability

The data associated with the paper are available from the corresponding author on reasonable request.

## Results

### Demographic and clinical results

Overall, 505 patients underwent an EVAR-procedure between January 2002 and August 2019; in 103 patients (20.4 %) a type II endoleak was identified on both 3 months and 1-year follow-up CTA. The vast majority of the study population were men (n = 98, 95%) with mean age 74 years who underwent EVAR with use of an Excluder stent-graft (W.L. Gore and Associates, Flagstaff, AZ, USA) (n = 41, 40%) as summarized in Table 1.

**TABLE 1.** Patients’ and procedural characteristics

**Clinical characteristic**	
Age (years)	
Mean age	74 (min 58; max 92)
Sex	
Female	n = 5 (5%)
Male	n = 98 (95%)
**Risk factors for atherosclerotic aortic disease according to Doyle *et al*.^11^**	
ASA-classification	
ASA 1	n = 4 (3.8%)
ASA 2	n = 18 (17.3%)
ASA 3	n = 75 (72.1%)
ASA 4	n = 6 (5.7%)
Cardiac disease	n = 49 (47.1%)
Carotid disease	n = 16 (15.4%)
Hyperlipidemia	n = 84 (80.7%)
Renal insufficiency	n = 43 (41.3%)
Diabetes mellitus	n = 26 (25%)
Arterial hypertension	
Controlled	n = 83 (79.8%)
Uncontrolled	n = 8 (7.7%)
Pulmonary disease	n = 38 (36.5%)
Smoking	
Active	n = 9 (8.6%)
Previous	n = 74 (71.1%)
**Stent-graft for EVAR**	
Excluder	n = 41 (40%)
Endurant	n = 20 (19%)
Ovation	n = 23 (22%)
Zenith	n = 13 (13%)
Quantum	n = 4 (4%)
Other	n = 2 (2%)

ASA = American Society of Anesthesiology; EVAR = endovascular aortic repair

In the follow-up period, 38 out of 103 patients (37%) with a persistent type II endoleak underwent treatment, including percutaneous embolization (n = 37; 97.3%), conversion to open repair (n = 1; 2.7%). The mean time interval between the index EVAR procedure and the treatment for persistent type II endoleak, associated to aneurysm sac expansion was 1370 days (range: 236–4173 days).

### Computed tomography angiography data

Maximum Ax and Per diameter and volume of the excluded aneurysm sac at 3 months and 1 year after EVAR as well as absolute and relative changes in Ax and Per diameter and volume between measurements on CTA at 3 months and 1 year after EVAR are summarized in Table 2.

**TABLE 2.** Diameter and volume measurements of the excluded aneurysm sac of 103 patients

**Maximum diameter / volume**	
Axial diameter (mm) at 3 months	63.7 (min 36.0; max 138.0)
Axial diameter (mm) at 1 year	63.4 (min 37.0; max 133.0)
Perpendicular diameter (mm) at 3 months	63.2 (min 35.0; max 141.0)
Perpendicular diameter (mm) at 1 year	63.0 (min 36.0; max 142.0)
Volume aneurysm sac (cm^3^) at 3 months	213.8 (min 67.0; max 1316.0)
Volume aneurysm sac (cm^3^) at 1 year	209.3 (min 72.0; max 1147.0)
Axial diameter absolute change (mm)	−0.3 (min −20.0; max −7.0)
Axial diameter relative change (%)	−0.51 (min −30.8; max 8.7)
Perpendicular diameter absolute change (mm)	−0.2 (min −20.0; max −7.0)
Perpendicular diameter relative change (%)	−0.2 (min −30.8; max 10.5)
Volume absolute change (cm^3^)	−4.5 (min −169.0; max −55.0)
Volume relative change (%)	−1.6 (min −50.7; max 20.8)

**FIGURE 1.**
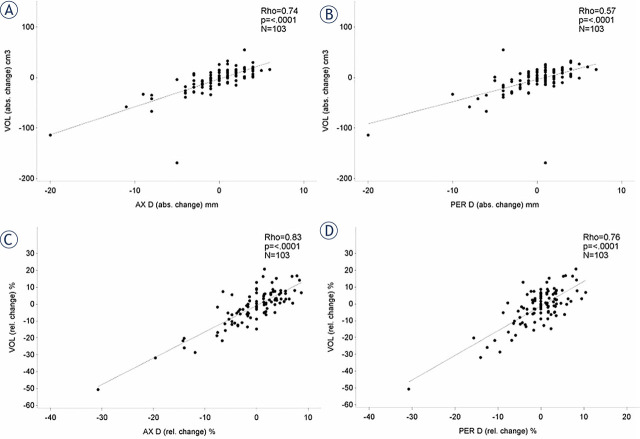
Correlation between aneurysm sac maximum axial or perpendicular diameter and volume at 3 months and 1 year with ϼ-values of 0.89, 0.90 and 0.91, 0.92 respectively; correlations between absolute differences are 0.74 **(A)**, 0.57 **(B)** and 0.83 **(C)**, 0.76 **(D)** respectively.

Correlation between Ax / Per diameter and volume measurements at 3 months and 1 year of follow-up are summarized in Table 3 and Figure 1, showing excellent correlation between aneurysm sac maximum Ax or Per diameter and volume at 3 months and 1 year with ϼ values of 0.89, 0.90 and 0.91, 0.92 respectively; correlations between absolute differences are 0.74, 0.57 and 0.83, 0.76 respectively.

**TABLE 3.** Correlation between axial / perpendicular diameter and volume at 3 months and 1 year

**At 3 months of follow-up**			
**Association of sac volume (cm^3^) with**	**Rho**	**95% CI**	**P-value**
Axial diameter (mm)	0.89	(0.84; 0.92)	< 0.0001
Perpendicular diameter (mm)	0.90	(0.86; 0.93)	< .0001
**At 1 year of follow-up**			
**Association of sac volume (cm^3^) with**			
Axial diameter (mm)	0.91	(0.87; 0.93)	< 0.0001
Perpendicular diameter (mm)	0.92	(0.89; 0.95)	< 0.0001
**Association of volume (absolute changes) (cm^3^) with**			
Axial diameter (mm)	0.74	(0.64; 0.82)	< 0.0001
Perpendicular diameter (mm)	0.57	(0422; 0.69)	< 0.0001
**Association of volume (relative changes) (cm^3^) with**			
Axial diameter (mm)	0.83	(076; 0.89)	< 0.0001
Perpendicular diameter (mm)	0.76	(0.66; 083)	< 0.0001

Finally, the potential agreement between diameter increase and volume increase above a threshold of 5 mm and 12% respectively, as proposed by Quan *et al*.,^12^ were tested and summarized in Table 4. In patients with an underlying type II endoleak, we did not find evidence of agreement between a diameter increase > 5 mm and a sac volume increase > 12% with a κ-value of 0.032 and 0.121 respectively between volume and Ax diameter and volume and Per diameter.

**TABLE 4.** Agreement between axial (Ax)/perperdicular (Per) sac diameter and sac volume

**Diameter**	**statistic volume change < 12%**	**volume change > 12%**
**Ax diameter**		
< 5 mm change	n/N (%) 93/95 (97.9%)	8/8 (100%)
> 5 mm change	n/N (%) 2/95 (2.1%)	0/8 (0%)
**Per diameter**		
< 5 mm change	n/N (%) 92/95 (96.8%)	7/8 (87.5%)
> 5 mm change	n/N (%) 3/95 (3.2%)	1/8 (12.5%)

### Correlations between computed tomography angiography data and later intervention for type II endoleak

Ax / Per diameter and volume changes in patient subgroup with and without later intervention for type II endoleak management are summarized in Table 5, showing no evidence of an association between the diameter or volume change and later need for intervention.

**TABLE 5.** Diameter and volume changes in patients with (n = 27) and without (n = 76) later intervention for type II endoleak

**Diameter / volume changes between 3 months and 1 year of follow-up**			
	**no intervention**	**intervention**	**P-value**
Ax diameter absolute change (mm)	−0.22 (min −9.0; max 6.0)	−0.48 (min −20.0; max 5.0)	0.37
Ax diameter relative change (%)	−0.43 (min −14.3; max 8.7)	−0.74 (min −30.8; max 7.8)	0.37
Per diameter absolute change (mm)	0.05 (min −10.0; max 7.0)	−0.85 (min −20.0; max 5.0)	0.91
Per diameter relative change (%)	0.15 (min −15.6; max 10.4)	−1.3 (min −30.8; max 8.2)	0.79
Volume absolute change (cm^3^)	−2.22 (min −67.0; max 55.0)	−10.89 (min −169.0; max 33.0)	0.96
Volume relative change (%)	−1.4 (min −28.6; max 14.2)	−2.2 (min −10.3; max 7.4)	0.92

Ax = axial; Per = perpendicular

## Discussion

In this study on 103 patients presenting with a type II endoleak after EVAR as identified by follow-up CTA, an excellent agreement between maximum aneurysm sac axial and perpendicular diameter measurement and sac volume measurement was found, with ϼ-values in between 0.84 and 0.95. This observation is in line with several studies^8,9,13^, but in contradiction to other studies.^7,14,15^ In the presented study, we tested the hypothesis by Quan *et al*.^12^, showing a diameter increase cut-off of 5 mm correlates to a volume increase of 12%; however, this observation could not be confirmed by this study. In the presented study, we only included patients with type II endoleaks as this subgroup of patients is at higher risk for late adverse outcomes after EVAR.^2^ However we did not perform a comparative study between patients with and without type II endoleak. In addition, a good correlation was found between change in maximum Ax / Per diameter and volume measurement between 3 months and 1 year of follow-up after EVAR with ϼ-values 0.57 and 0.83. Here a better correlation for Ax diameter versus Per diameter to volume measurement was observed. Adversely, Boos *et al*., including both patients with and without type II endoleaks, found an increase in centreline diameter (= perpendicular diameter) and volume (measured from the lowest renal artery to the iliac bifurcation) as most sensitive criteria for detecting endoleaks.^8^ Schnitzbauer *et al*. found a low to moderate sensitivity for the detection of volume increase compared to diameter measurements with cut-off values of > 5 mm and > 5%; however these authors did not analyze absolute or relative changes in diameter versus volume as predictors for type II endoleak.^7^

This study could not demonstrate differences in early changes in aneurysm sac diameter nor volume in patients who needed or did not need endoleak-related re-intervention in a later phase. Therefore, continued follow-up including contrast-enhanced CTA is still mandatory to identify endoleak volume or diameter growth or changes in endoleak pattern as demonstrated by Dudeck *et al*.^5^ In addition, combined findings of type II endoleak associated with clear growth of the sac diameter > 1 cm are mostly found later than 1 year after index EVAR procedure.^4^

Limitations of this study are multiple. First, different CT-scanners with different scan protocols were used, related to the long-time interval included patients were scanned after their EVAR procedure. However, scan and injection protocols did not change significantly between 16-, 64- and 256-row MDCT.^16^ Second, no intranor interobserver variability studies on diameter and volume measurements were performed and all measurements were performed in consensus by two radiologists. However, acceptable intra- and interobserver variability of aortic aneurysm volume measurement with or without semi-automated tools has been demonstrated by van Prehn *et al*..^17^ Third, various types of endografts were used; nevertheless, sac measurements on CTA or indications for re-intervention are independent of the type of endograft. Fourth, only two sets of early follow-up CTA were included in the study; potentially, inclusions of measurements on CTA’s at two or more years of follow-up after EVAR might better select patients with type II endoleak for re-intervention as indication for type II-related intervention is made after a mean of 3 years postoperatively.^4^

In conclusion, this study demonstrates an excellent correlation of diameter and volume measurements of the aneurysm sac in patients with type II endoleak early after EVAR. However, these early changes in sac diameter or volume on CTA at 3 months and 1 year after initial EVAR cannot predict patients at later risk for type II endoleak-related re-intervention. Continued CTA is still needed to further monitor patients with type II endoleak after EVAR and eventually to select patients at risk for re-intervention.
